# Cigarette smoking and tuberculosis in Cambodia: findings from a national sample

**DOI:** 10.1186/1617-9625-11-8

**Published:** 2013-03-27

**Authors:** Pramil N Singh, Daravuth Yel, They Kheam, Glorietta Hurd, Jayakaran S Job

**Affiliations:** 1 Center for Health Research, School of Public Health, Loma Linda University, Phnom Penh, Cambodia; 2 World Health Organization/Tobacco Free Initiative, Phnom Penh, Cambodia; 3 National Institute of Statistics, Ministry of Planning, Phnom Penh, Cambodia; 4 Department of Epidemiology, Biostatistics, and Population Medicine, School of Public Health, Loma Linda University, Loma Linda, USA; 5 Department of Global Health, School of Public Health, Loma Linda University, Loma Linda, USA

## Abstract

**Background:**

Cambodia has very high rates of tuberculosis and smoked tobacco use among adults. Efforts to control both tobacco use and tuberculosis in Cambodia need to be informed by nationally representative data. Our objective is to examine the relation between daily cigarette smoking and lifetime tuberculosis (TB) history in a national sample of adults in Cambodia.

**Methods:**

In 2011, a multi-stage, cluster sample of 15,615 adults (ages 15 years and older) from all regions of Cambodia were administered the Global Adult Tobacco Survey by interviewers from the National Institute of Statistics of Cambodia.

**Results:**

Our findings include: 1) among daily smokers, a significant positive relation between TB and number of cigarettes smoked per day (OR = 1.70 [95% CI 1.01, 2.87]) and pack-years of smoking (OR = 1.53 [95% CI 1.05, 2.25]) 2) a non-significant 58% increase in odds of ever having being diagnosed with TB among men who smoked manufactured cigarettes (OR = 1.58 [95% CI 0.97, 2.58]).

**Conclusion:**

In Cambodia, manufactured cigarette smoking was associated with lifetime TB infection and the association was most evident among the heaviest smokers (> 1 pack per day, > 30 pack years).

## Introduction

Tuberculosis (TB) is the second most deadly infectious disease [1]. Global initiatives have set goals to reduce tuberculosis rates by half by 2015 through improved detection and treatment [2]. Recently, Basu et al. [2] noted that the millennium goals for TB cannot be achieved in high risk regions (Africa, South East Asia, Western Pacific Region) without major gains in reducing the number of current and future smokers. A causal pathway linking smoked tobacco to TB has been strongly supported by histopathologic evidence of lung damage in smokers that increases their vulnerability to deep lung infections [3].

During the past six decades, population-based evidence has accumulated that attributes a major TB burden to smoked tobacco [1,4]. Specifically, early evidence linking smoked tobacco to TB came from samples of high risk adults (i.e. health care workers, migrants, patients, elderly, prisoners) [5-7]. Recently, in a 2008 case control study of 1.1 million households in India, Jha et al. found that the TB death rate was 1.7 times higher in smokers as compared to nonsmokers [8]. In 2009, Jee et al. reported a significant 60% increase in risk of death among 1,294,504 South Korean adults [9], and a similar association with incident TB in men [9]. In the Taiwan National Health Interview Survey (n = 17,699, ≥ 12 y and older), Lin et al. [4] found a significant two-fold increase in risk of incident, active TB among current smokers [4].

In the present study, we examined the relation between cigarette smoking and TB infection in the 2011 National Adult Tobacco Survey of Cambodia (2011 NATSC). This was the largest national prevalence survey of adult tobacco use (n = 15,615, ages 15 years and older) conducted to date [10,11] and was completed as part of a Fogarty/NIH funded (Loma Linda University; National Institute of Statistics, Ministry of Planning (Cambodia); WHO Cambodia; Southeast Asia Tobacco Control Alliance) research capacity building program. For this analysis our specific aims are as follows: 1) To examine the relation between daily cigarette smoking and TB 2) To examine the relation between manufactured cigarette smoking and TB 3) To examine whether the intensity of cigarette smoking (pack-years, number of cigarettes smoked) among daily smokers further increased the likelihood of developing TB.

## Methods

### Study population

Using the 2008 census as a sampling frame, the 2011 NATSC sample was selected using a stratified, multi-stage cluster sample described in detail elsewhere [10,11]. Briefly, Cambodia was stratified into 17 census-derived survey domains composed of 12 individual provinces and 5 groups of similar provinces. For the first stage of sampling, 25–26 primary sampling units (PSU) were selected from each domain (i.e. villages or comparable urban unit). There were a total of 437 PSUs surveyed. In the second stage, a circular systematic sampling method was used to select 12 households from every urban PSU and 15 households from every rural PSU. A total of 86 interviewers and enumerators were trained by the National Institute of Statistics and three of the report authors (DY, TK, PNS) during a one-week session in Phnom Penh that preceded the data collection efforts.

The sampling method resulted in 15,615 adults (ages 15 years and older) selected from 6,294 households inclusive of all private and single member households from all provinces. The survey did not include institutional households such as military barracks, prisons, hospitals, and residents of temples.

Written informed consent was obtained from each subject and an incentive provided for participation (US$ 0.50). The study protocol was approved by the Institutional Review Board of Loma Linda University and the National Ethics Committee on Health (Ministry of Health) in Cambodia.

### Questionnaire

The questionnaire for the 2011 NATSC was designed based on 1) qualitative studies to determine items on tobacco use and other lifestyle variables and obtain representative pictures for pictograms [12] 2) standardized items of the Global Adult Tobacco Survey (GATS) [13] 3) the 2006 national survey of tobacco use in Cambodia [10]. The final survey contained sections on demographics, smoked tobacco, smokeless tobacco, cessation, secondhand smoke, economics, media, knowledge, attitudes and perceptions, diet, current health/access to health care, and women’s health. In the section on current health, subjects provided a self-report of infectious disease (tuberculosis, HIV/AIDS, malaria) status using an item that asked “Has a doctor or other health worker EVER diagnosed or told you that you are suffering from (infectious disease)?”

Translation of the survey (English to Khmer) was accomplished using methods described by Flaherty [14]. Data entry and quality control was accomplished using the Census and Survey Processing System (CSPro; Suitland, MD).

### Statistical analysis

The smoked tobacco use exposure variables used in the analysis were created using a standardized coding method for GATS items. To examine the relation between smoked tobacco use as an exposure and self-reported tuberculosis as an outcome we used a multivariable logistic regression model. Age and other pertinent confounders (gender, Second hand smoke, education, rural dwelling, and income) were also tested and retained if they substantially affected the exposure. The continuous measures of intensity (number of cigarettes, years smoked, pack-years) were modeled by log (to base *e*) transforming each variable *x* such that the relation between odds of TB and *x* could be described as:

$$\mathrm{OR}=e^{\beta \text{log}x}=x^{\beta }$$

where a linear relation is modeled for β = 1, exponential relation for β > 1, and root function (allowing threshold effects) for β < 1 [15]. This more flexible set of functions allows a hypothesized increase or decrease in odds of TB to follow curvilinear positive or negative trends. Non-linear trends were tested using spline regression [15].

To account for the stratified, multi-stage cluster design, the variance for calculating 95% confidence intervals for measures of effect (odds ratios), prevalence, and means were computed using a Taylor series linearized method that accounted for between and within cluster correlation. Point estimates were further adjusted by sample weights. Statistical analyses were preformed with SUDAAN software release 9.0 (RTI International, Research Triangle Park, NC, USA).

## Results

Among NATSC 2011 subjects (n = 15,615) we found a prevalence of ever having been diagnosed with Tuberculosis (TB) of 1.19% [95% CI = 0.93 to 1.52]. Using the census derived sampling weights, we computed that this weighted prevalence estimate represents 88,859 cases [95% CI = 67,696 to 110,018] of lifetime TB infection among adults ages 15 years and older during 2011. In Table 1, we examined the how TB prevalence and number of estimated cases varies by category of demographic variables. The rate of TB approximately doubled between adolescence (age 15–17 years) and age ≥ 45 years. A higher burden of TB was found for adults who were married or in cohabitation (est. 68,674 cases), rural adults (est. 83,945 cases), adults earning < 1 USD per day (est. 52,358), and those with < 6 y of education (est. 74,713 cases). By occupation, we found that most of the TB cases were occurring in agricultural workers (est. 63,886 cases). An unexpected finding was the very high prevalence of TB among traditional healers (14.2% among all traditional healers; 36.7% among male traditional healers).

**Table 1:** Prevalence of lifetime history of Tuberculosis [95% CI] and estimated number of cases of lifetime TB infection are given by demographic variables among 15,615 subjects of the 2011 National Adult Tobacco Survey of Cambodia (NATSC 2011)

	**Total**	**Men**	**Women**
	**Prevalence [95% CI] estimated number of cases**	**Prevalence [95% CI] estimated number of cases**	**Prevalence [95% CI] estimated number of cases**
**Age**			
≥15 and ≤17	0.73[0.36, 1.50]	0.96[0.36,2.55]	0.50[0.20,1.24]
	4745	3128	1617
>17 and ≤24	0.38[0.21, 0.69]	0.22[0.08,0.59]	0.54[0.26,1.10]
	4754	1356	3398
>24 and <45	1.11[0.79, 1.56]	1.31[0.88,1.95]	0.96[0.64,1.43]
	33545	17491	16053
≥45 and <65	1.78[1.31,2.40]	1.62[1.05,2.49]	1.89[1.34,2.64]
	35225	13390	21835
≥65	1.88 [1.11, 3.16]	1.74[0.81,3.73]	2.01[1.10,3.62]
	10587	4639	5947
**Ethnicity**			
Khmer	1.18[0.92,1.51]	1.16[0.83,1.62]	1.19[0.93,1.52]
	85043	37931	47113
Cham	0.86(0.23,3.17)	0.21[0.02,1.80]	1.39[0.27,6.74]
	1001	108	894
Chinese	2.58[0.90,7.17]	3.95[1.50,10.00]	1.43[0.39,5.17]
	2812	1966	846
**Religion**			
Buddhist	1.19[0.93,1.52]	1.18[0.85,1.64]	1.19[0.93,1.53]
	86356	38718	47637
Muslim	0.88[0.24,3.20]	0.21[0.02,1.80]	1.44[0.29,6.91]
	1001	108	894
Other	2.58[0.91,7.08]	4.17[1.42,11.64]	1.13[0.36,3.48]
	1392	1071	321
None	0.96[0.17,5.21]	1.72[0.31,8.92]	_
	108	108	
**Marriage**			
Never married	0.46[0.28,0.78]	0.51[0.26,1.00]	0.42[0.21,0.82]
	8990	5106	3884
Currently Married	1.34[1.03,1.75]	1.49[1.08,2.06]	1.21[0.91,1.62]
	63149	32835	30315
Live together	2.49[1.34,4.60]	_	2.99[1.60,5.52]
	5525		5525
Widower/widow	1.77 [1.05,2.94]	1.25[0.31,4.92]	1.85[1.07,3.21]
	8847	922	7925
Divorced	2.62[0.69, 9.43]	2.67[0.46,13.95]	2.59[0.86,7.50]
	2346	1142	1203
**Education**			
0 years	2.03[1.52,2.71]	1.73[1.07,2.80]	2.16[1.56,2.97]
	35060	8619	26441
(>0-6 y)	1.25[0.93,1.69]	1.55[1.07,2.24]	1.00[0.71,1.41]
	39653	22567	17085
(7–9 Y)	0.50[0.28,0.89]	0.53[0.26,1.09]	0.46[0.21,1.02]
	8352	4830	3521
(10–12 Y)	0.68[0.34,1.38]	0.87[0.44,1.75]	0.44[0.15,1.31]
	5310	3805	1505
(>12 Y)	0.36[0.11,1.18]	0.28[0.04,1.96]	0.49[0.12,2.03]
	377	184	193
**Income**			
<1 USD	1.26(0.96,1.65)	1.25[0.84,1.86]	1.27[0.96,1.67]
	52358	18303	34055
1-2 USD	1.07[0.66,1.74]	1.05[0.59,1.86]	1.09[0.53,2.24]
	10451	5352	5099
>2-3 USD	1.27[0.67,2.39]	1.28[0.61,2.64]	1.26[0.62,2.53]
	10662	6033	4629
>3 USD	1.04[0.68,1.57]	1.12[0.68,1.84]	0.90[0.42,1.91]
	15387	10316	5069
**Occupation**			
No Occupation	1.09[0.71,1.67]	0.95[0.47,1.89]	1.17[0.73,1.87]
	13498	3952	9546
Professional	0.95[0.32,2.77]	1.35[0.45,3.96]	_
	839	839	
Traditional Healer/Faith Healer	14.19[1.55,63.52]	36.71[3.47,90.34]	_
	586	586	
Technician other Professional	0.08[0.01,0.59]	_	0.30[0.04,2.12]
	91		91
Service Workers	0.47[0.06,3.33]	_	0.77[0.10,5.48]
	184		184
Fireman, Police, other protective Services	1.20[0.17,8.13]	1.32[0.18,8.93]	_
	403	403	
Sales	0.48[0.23,0.99]	0.22[0.07,0.70]	0.58[0.26,1.26]
	3077	381	2695
Farming Livestock	1.44 [1.11,1.87]	1.34[0.95,1.87]	1.53[1.16,2.01]
	63887	27843	36043
Labor	0.90[0.39,2.09]	1.15[0.48,2.77]	0.20[0.03,1.44]
	4939	4647	293
Trades and Crafts	0.23[0.03,1.56]	1.10[0.16,7.27]	_
	514	514	
Armed Forces	4.00[0.86,16.71]	4.13[0.88,17.24]	_
	840	840	
**Rural/Urban**			
Urban	0.39[0.19,0.79]	0.52[0.23,1.19]	0.29[0.12,0.69]
	4912	2846	2066
Rural	1.36[1.05,1.75]	1.32[0.93,1.85]	1.39[1.08,1.78]
	83945	37159	46785
**Alcohol**			
No Alcohol	1.14[0.89,1.46]	1.18[0.83,1.67]	1.12[0.86,1.45]
	65,290	23,653	41,638
Beer	1.05[0.52,2.13]	1.04[0.47,2.27]	1.09[0.37,3.15]
	6691	5477	1214
Wine	1.31[0.71,2.41]	1.35[0.68, 2.68]	1.13[0.30, 4.13]
	7233	6077	1155
Spirits	1.53[0.95,2.47]	1.21[0.65, 2.23]	2.67[1.32,5.31]
	12164	7461	4703
Palm Liquor	1.83[0.63, 5.14]	0.78[0.19,3.15]	3.85[1.04,13.25]
	1748	493	1255

### Univariate associations between smoked tobacco, demographics, and TB

In Table 2, we provide the univariate models relating smoked tobacco, manufactured cigarette smoking, and pertinent demographic variables to odds of having ever been diagnosed with TB. For smoked tobacco, we found non-significant increases (30-42%) in odds of TB among all subjects and among men – the primary users of smoked tobacco in Cambodia.

**Table 2:** Univariate odds ratios relating smoked tobacco, manufactured cigarette smoking, and demographics to tuberculosis among 15,615 subjects of the 2011 National Adult Tobacco Survey of Cambodia

	**All subjects**	**Men**
	**OR [95% CI]**	**OR [95% CI]**
**All Smoked Tobacco**		
Current Smoker	1.30[0.89,1.89]	1.42[0.90, 2.24]
Non-smoker	1.00 [referent]	1.00 [referent]
**Manufactured Cigarette**		
Current Smoker	1.31[0.84,2.05]	1.58[0.97,2.58]
Non-smoker	1.00 [referent]	1.00 [referent]
**Demographics**		
Age (per 1 year)	1.02[1.01,1.03]	1.02[1.01,1.03]
Gender		
Female	1.01[0.75,1.34]	-
Male	1.00 [referent]	
Residence		
Rural	3.51[1.63,7.58]	2.55[1.03,6.27]
Urban	1.00 [referent]	1.00 [referent]
Daily exposure to Environmental Tobacco Smoke		
Yes	1.11[0.71,1.76]	0.98[0.56,1.73]
No	1.00 [referent]	1.00 [referent]
Education		
0 years	1.00 [referent]	1.00 [referent]
0 -6 years	0.61[0.43,0.86]	0.89[0.55,1.46]
7-9 years	0.24[0.13,0.44]	0.30[0.14,0.65]
10-12 years	0.33[0.16,0.68]	0.50[0.23,1.11]
> 12 years	0.17[0.05,0.59]	0.16[0.02,1.17]
Daily Income		
< 1 USD	1.00 [referent]	1.00 [referent]
1-2 USD	0.85[0.54,1.34]	0.84[0.47,1.50]
>2-3 USD	1.01[0.53,1.91]	1.02[0.48,2.19]
>3 USD	0.82[0.52,1.30]	0.89[0.50,1.59]

The relation between manufactured cigarette smoking and TB was particularly evident in men (OR = 1.58 95% CI [0.97, 2.58]). The lifetime TB prevalence and estimated number of cases of lifetime TB infection per 10,000 was higher for manufactured cigarette smokers (1.60 95% CI [0.99, 2.56]; 160 cases per 10,000 men) than for non-smokers (1.02 95% CI [0.72, 1.43]; 102 cases per 10,000 men). Thus, manufactured cigarettes were contributing to an annual excess of 58 TB cases per 10,000 men.

Among the demographic variables we found the expected positive association with age indicating a significant 2% increase in odds of TB per year of age during adulthood. Rural residence was strongly associated with increased odds of TB (OR = 3.51 [95% CI 1.63, 7.58]. Higher education (> 12 years) was associated with a more than five-fold decrease in odds of TB (OR = 0.17 95% CI [0.05, 0.59] relative to no schooling. Taken together, these data indicate that rural lifestyle patterns may be strong predictors of TB.

### Tuberculosis and intensity of smoking habit among daily smokers

In Table 3, we examined whether the intensity of smoking further increased the odds of TB among daily smokers and considered three log-transformed measures of intensity: 1) number of cigarettes smoked per day 2) length of smoking habit 3) pack-years of cigarette smoking. These findings indicated that, among daily smokers, significant positive trends were found for number of cigarettes smoked per day and pack years. Moreover, these findings remained evident and even slightly stronger in multivariable analysis. Non-linear were tested using spline regression but were not evident.

**Table 3:** Odds ratios relating intensity of smoking habit to tuberculosis among 2,629 daily smokers of the 2011 National Adult Tobacco Survey of Cambodia

	**Age-adjusted**	Multivariable*
	**OR [95% CI]**	**OR [95% CI]**
**Log (Number of Cigarettes)**	1.70 [1.01, 2.87]	1.74 [1.01, 2.99]
**Log (Years of Smoked)**	1.45 [0.68, 3.06]	1.43 [0.68, 3.02]
**Log (Pack Years)**	1.53 [1.05, 2.25]	1.56 [1.04, 2.32]

*adjustment for age, rural dwelling, weekly consumption of beer, wine, spirits (excluding homemade palm liquor), homemade palm liquor.

**Figure 1:**
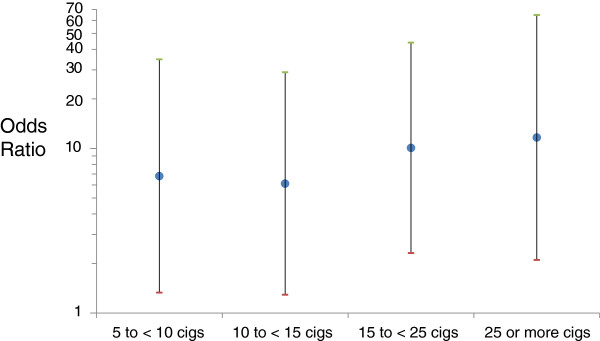
Age-adjusted odds ratios for tuberculosis are presented for categories of number of cigarettes smoked among 3,091 daily smokers of the 2011 National Adult Tobacco Survey of Cambodia.

**Figure 2:**
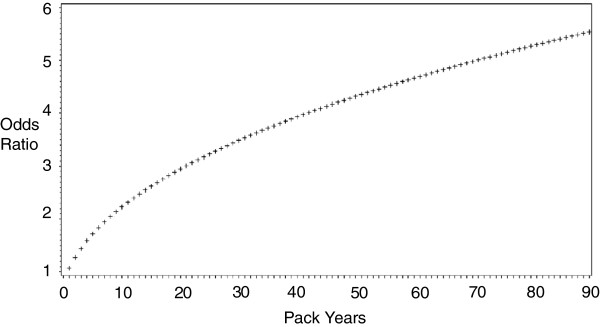
Fitted odds ratios of TB derived from age-adjusted logistic regression model with a log transformed variable for pack years among daily smokers of the 2011 National Adult Tobacco Survey of Cambodia*. *referent categories for number of cigarettes and pack years were 2.5 cigarettes and 0.7 pack-years, respectively.

In Figures 1, 2 we provide odds ratios for common values of number of cigarettes and pack-years (OR for pack years solved from the log transformed model) for ease of interpretation. In Figure 1, we modeled categories of number of cigarettes smoked and found that a smoking habit of at least a pack a day was associated with a more than 10-fold increase in odds of TB relative to < 5 cigarettes per day (OR [95% CI] for cigarettes/day = 1.00 [referent] for < 5; 6.79 [1.33, 34.77] for 5 to < 10; 6.11 [1.29, 29.01] for 10 to < 15; 10.07 [2.31, 43.89] for 15 to <25; 11.65 [2.10, 64.57] for ≥ 25). In Figure 2, we plot the association between the odds ratio for TB and log-transformed pack-years.

Also, noteworthy is that the association between length of smoking habit and TB given in Table 3, was much stronger when analyses were restricted to smokers of manufactured cigarettes (OR = 2.02 [0.91, 4.48]).

### Multivariable models

In multivariable models, the addition of covariates (rural residence, Second hand smoke exposure, education, alcohol, and income) in addition to age did not substantially alter the measures of effect for the tobacco variables.

## Discussion

We examined the association between cigarette smoking and tuberculosis (TB) among 15,615 adults (ages 15 years and older) enrolled in the largest survey of adult tobacco use ever conducted in Cambodia [10,11]. Our major findings include: 1) a non-significant 58% increase in odds of ever having being diagnosed with among men who smoked manufactured cigarettes (OR = 1.58 [95% CI 0.97, 2.58]) 2) Among daily smokers, a significant positive relation between TB and log transformed variables for number of cigarettes smoked per day (OR = 1.70 [95% CI 1.01, 2.87]) and pack-years of smoking (OR = 1.53 [95% CI 1.05, 2.25].

Our findings from a large, representative national sample of Cambodia add to the evidence from India [8], South Korea [9], Taiwan [4], Thailand [3], and Malaysia [16] that identify smoked tobacco as a major contributor to the TB burden in Asia. Taken together with meta-analyses from small samples from 19 nations [5-7], and recent global estimates from mathematical modeling [2], these data support that the global reduction of tuberculosis infection is heavily dependent on successful tobacco control being achieved in Asia [17].

### Pathophysiology of smoking intensity and tuberculosis infection

Our findings identify a more than 3-fold increase in odds of TB among adults who were smoking one pack a day or more or those who had smoked greater than 30 pack-years (Figures 1, 2). These data on heavy smokers are concordant with much of what is known of the mechanism of increased susceptibility to TB infection in smokers [3,18]. Such smoking-induced mechanisms include: 1) an impairment of mucociliary function [19,20] 2) lower airway epithelial damage and inflammation [19,21] 3) a constriction of the alveolar airsac [17,19,22] 4) an increase in the number of circulating alveolar macrophages (the cells targeted by tuberculosis) [17,23]. 5) a collapse of the bronchioles [1,24-26]. Beyond physical changes, the immune suppression from heavy smoking could also contribute to TB infection of the lung [17,19,27,28].

### Cigarette smoking as a component of a Bio-behavioral framework linking TB infection with respiratory disease risk factors in rural adults of the western pacific region

The association between TB and smoking among the primarily rural adults of Cambodia that we studied, needs to be considered in the context of the many other environmental factors in this region that can contribute to TB infection. Specifically, much of the smoking-related lung damage described above that potentially increases risk of TB infection, can also be caused by the high rates of exposure to Second hand smoke [29,30], indoor cooking fires [31,32], crop-burning [32], and occupational dust and dirt that is highly prevalent in the region.

Also noteworthy are pathogen transmission pathways present in the rural lifestyle such as crowding in household environments and health and hygiene practices.

In our analysis, it is noteworthy that the two of the strongest demographic risk factors included rural residence and less years of education (Table 2). Among women and ethnic minorities of Cambodia and the region there is also a possible link between non-cigarette forms of tobacco (i.e. betel quid, waterpipe) and TB and/or lung damage [10,33,34].

### Implications for tobacco and tuberculosis control programs in Cambodia and the western pacific region

Our findings estimate that there is an excess of 58 TB cases per 10,000 Cambodian men due to the smoking of manufactured cigarettes (a baseline rate in non-smokers of 102 cases per 10,000 men). It is noteworthy that our 2011 findings indicate that manufactured cigarette smoking is the predominant form of smoked tobacco sold in Cambodia (18 out of 21 cigarettes sold are manufactured cigarettes) – a recent trend that is likely due to the lower price per pack (0.20 USD per pack) [11]. The current survey also indicated that 95% of the manufactured packs had a tax stamp [11] that can be used to set the price. Taken together, these findings indicate that implementation of WHO Framework Convention on Tobacco Control initiatives to increase the tax on these packs can be effective in not only controlling tobacco use but also in tuberculosis control initiatives. Since Cambodia has a very high prevalence of both tuberculosis (21^st^ in the world) and smoked tobacco habits, future efforts to coordinate tobacco and tuberculosis control programs should be considered. For example, the timing of national tuberculosis screening with FCTC implementation efforts (i.e. effective increases in the price of manufactured cigarettes) can measure the efficacy of a coordinated control effort.

### Limitations

Limitations of our analysis of this 2011 national sample of Cambodia need description. We have examined the relation between smoked tobacco and self-reported TB in a cross-sectional analysis and thus we cannot directly infer causation. The report of TB to our trained health interviewers was by an item that measured ever having been diagnosed with TB during a subject’s lifetime. Such a measure does not discriminate between active primary TB infection, active secondary TB infection, or Latent TB infection [35]. Also, despite controlling for a number of indicators of poverty, unmeasured confounders such as number of rooms per house and number of household members were not accounted for in the analysis. Lastly, findings of this study do not apply to institutionalized individuals and the tobacco-TB association would need further investigation in studies of these subgroups.

## Conclusion

Our findings from a large national sample of adults in Cambodia identify that a history of TB infection was more common among men who smoked manufactured cigarettes and the heaviest smokers (> 1 pack per day, > 30 pack years). The high prevalence of smoking and TB in Cambodia and the region, identifies a need for coordinating control measures for smoking and TB.

## Competing interests

The authors declare that they have no competing interests.

## Authors’ contribution

PNS and DY conceived the study, obtained the funding, analyzed the data, and, and drafted the report; TK directed the data collection and sampling; GH assisted in data analysis and editing of the report; JSJ obtained funding and edited the report. All authors’ read and approved the final manuscript.
